# Integrated Functions of Pax3 and Pax7 in the Regulation of Proliferation, Cell Size and Myogenic Differentiation

**DOI:** 10.1371/journal.pone.0004475

**Published:** 2009-02-16

**Authors:** Charlotte A. Collins, Viola F. Gnocchi, Robert B. White, Luisa Boldrin, Ana Perez-Ruiz, Frederic Relaix, Jennifer E. Morgan, Peter S. Zammit

**Affiliations:** 1 Dubowitz Neuromuscular Centre, UCL Institute of Child Health, London, United Kingdom; 2 King's College London, Randall Division of Cell and Molecular Biophysics, New Hunt's House, London, United Kingdom; 3 Mouse Molecular Genetics group, UMR S 787 - Groupe Myologie, INSERM - UPMC-Paris VI, Faculté de Médecine Pitié-Salpétrière, Paris, France; Baylor College of Medicine, United States of America

## Abstract

Pax3 and Pax7 are paired-box transcription factors with roles in developmental and adult regenerative myogenesis. Pax3 and Pax7 are expressed by postnatal satellite cells or their progeny but are down regulated during myogenic differentiation. We now show that constitutive expression of Pax3 or Pax7 in either satellite cells or C2C12 myoblasts results in an increased proliferative rate and decreased cell size. Conversely, expression of dominant-negative constructs leads to slowing of cell division, a dramatic increase in cell size and altered morphology. Similarly to the effects of Pax7, retroviral expression of Pax3 increases levels of *Myf5* mRNA and MyoD protein, but does not result in sustained inhibition of myogenic differentiation. However, expression of Pax3 or Pax7 dominant-negative constructs inhibits expression of *Myf5*, *MyoD* and *myogenin*, and prevents differentiation from proceeding. In fibroblasts, expression of Pax3 or Pax7, or dominant-negative inhibition of these factors, reproduce the effects on cell size, morphology and proliferation seen in myoblasts. Our results show that in muscle progenitor cells, Pax3 and Pax7 function to maintain expression of myogenic regulatory factors, and promote population expansion, but are also required for myogenic differentiation to proceed.

## Introduction

The *Pax* gene family contains nine members characterised by the presence of a common paired-box domain that directs binding to specific DNA sequences. *Pax* genes encode transcription factors that have important and highly conserved roles during development. In skeletal muscle, Pax3 and Pax7 have overlapping, but non-redundant roles in the specification of embryonic muscle progenitors, and network with the myogenic regulatory factor (MRF) family of transcription factors comprising Myf5, MyoD, Mrf4 and myogenin [reviewed in 1]. During the earliest stages of embryonic muscle development, *Pax3* and *Myf5* lie genetically upstream of *MyoD* but in later developmental stages both *Myf5* and *MyoD* function downstream of *Pax3* and *Pax7* [2], [3]. In C2C12 immortalised myoblasts, Pax7 has recently been shown to induce chromatin modifications through association with a histone methyltransferase complex and direct binding to regulatory regions of the *Myf5* locus [4].

In postnatal skeletal muscle, the primary cellular source of growth and regeneration is the satellite cell [5]–[7], a quiescent muscle precursor cell situated beneath the basal lamina that surrounds each muscle fibre. In response to muscle injury, satellite cells are activated, proliferate to form a pool of myoblasts, commit to differentiation and then fuse together to repair or replace damaged muscle fibres (reviewed, [8]). Pax7 is expressed almost ubiquitously by quiescent satellite cells and is co-expressed with MyoD in their proliferating myoblast progeny [9], [10]. Pax3 is transiently detected in proliferating satellite cell-derived myoblasts [11]–[13]. Furthermore, in different *Pax3* reporter lines, for example the *Pax3^eGFP/+^* mouse, activity at the *Pax3* locus is reported in a subset of muscles, in both quiescent and proliferating satellite cells [14], [15]. Pax7 is specifically required for maintenance of postnatal muscle. In the *Pax7^−/−^* mouse, satellite cells are present at birth in near-normal numbers but their population becomes rapidly depleted during the early postnatal period [15]–[18]. Myogenin is an early marker of commitment to differentiation and initiation of its expression occurs concomitantly with the down regulation of Pax7, which is subsequently absent from differentiated myonuclei [9], [10], [19]. In myoblast cell cultures Pax7 is similarly not expressed in differentiated myotubes, but is maintained in the smaller accompanying population of undifferentiated cells that stops proliferating, down regulates MyoD and returns to a non-proliferating state reminiscent of the quiescent satellite cell [10]. The precise influences of Pax7 and Pax3 on myogenic progression remain a subject of debate. In C3H10T1/2 cells converted to a myogenic phenotype by transduction with a MyoD vector, it has been shown that Pax7 and myogenin can regulate each other in a reciprocal manner, such that overexpression of Pax7 prevents myogenin induction and overexpression of myogenin causes Pax7 to be down regulated [20]. Accordingly, sustained retroviral expression of Pax7 causes a delay in myogenic differentiation in primary myoblasts [21]. Transfection of C2C12 cells [22] or primary myoblasts [23], [24] with Pax3 encoding constructs has variously been reported to inhibit differentiation [22], [23] or to be compatible with myotube formation [24].

Here, we show that the rate of cell division is influenced by Pax3 and Pax7. High-levels of *Pax* gene activation increase proliferative rate and prevents precocious myogenic differentiation. However, expression of Pax3 or Pax7 dominant-negative constructs results in a down regulation of Myf5, MyoD and myogenin, and prevents myogenic differentiation from proceeding. These findings suggest that in adult muscle stem cells, *Pax* genes function to promote population expansion, whilst maintaining commitment to the myogenic lineage.

## Materials and Methods

### Cell culture

C2C12 and NIH 3T3 cells were maintained in Dulbecco's modified Eagle's medium (DMEM) (Invitrogen) supplemented with 10% (v/v) foetal calf serum, 400 mM L-Glutamine (Sigma) and 1% (v/v) penicillin/streptomycin solution (Sigma). For differentiation studies, myogenic cells were cultured to confluency, at which point they began to spontaneously differentiate and fuse into myotubes. When analysed at least five days later, there were many large, multinucleated myotubes present.

### Single myofibre isolation

Mice were bred, and experimental procedures were carried out, in accordance with the Animals (Scientific Procedures) Act 1986. C57 Bl/10 wild type mice (aged 8–12 weeks) were killed by cervical dislocation and the extensor digitorum longus (EDL) muscle carefully dissected. Muscles were digested in 0.2% Collagenase Type 1 and individual myofibres dissociated by trituration and washed as described in detail elsewhere [25].

### Culture of myofibres and satellite cell-derived primary myoblasts

For suspension culture, myofibres were incubated in plating medium [DMEM with 10% (v/v) horse serum (PAA Laboratories), 0.5% (v/v) chick embryo extract (ICN Flow), 400 mM L-Glutamine (Sigma) and 1% (v/v) penicillin/streptomycin solution (Sigma)] at 37°C in 5% CO_2_. For adherent cultures, isolated myofibres were placed in 6-well plates (Nunc) coated with 1 mg/ml Matrigel (Collaborative Research). Plating medium was added and the cultures maintained at 37°C in 5% CO_2_. After 48 h in culture, myofibres were removed, and the remaining satellite cell-derived myoblasts trypsinised and re-plated in Matrigel-coated LAB-TEK® 8-well chamber slides (Nunc) with growth medium [DMEM supplemented with 20% (v/v) foetal calf serum, 10% (v/v) horse serum, 1% (v/v) chick embryo extract and 400 mM L-Glutamine (Sigma) and 1% (v/v) penicillin/streptomycin solution (Sigma)].

### Retroviral expression vectors

The retroviral backbone *pMSCV-puro* (Clontech) was modified to replace the puromycin selection gene with *eGFP*, to create *pMSCV-IRES-eGFP*, which served as the control vector. The generation of *pMSCV-Pax7-IRES-eGFP* using murine *Pax7d* (accession NM_011039) cDNA (Pax7 RV) has been described previously [21]. Murine *Pax3* cDNA (accession NM_008781) was cloned in *pMSCV-IRES-eGFP* to generate *pMSCV-Pax3-IRES-eGFP*, producing *Pax3* as a bicistronic message with *eGFP* (Pax3 RV). The Pax7 and Pax3 dominant-negative constructs have the N-terminus of Pax7 (first 340 amino acids) or Pax3 (374 amino acids), that directs DNA binding and so target gene selection, fused in frame with the active repression domain of *Drosophila* Engrailed, producing fusion proteins termed Pax7DN and Pax3DN respectively [15]. We previously confirmed that the Pax3DN and Pax7DN constructs were functional using cells from a Pax reporter line, *P34*, in which β-gal is expressed from an *nlacZ* reporter that is regulated by multimerized Pax3/7 binding sites [15], [26]. *Pax7DN* and *Pax3DN* were cloned in *pMSCV-IRES-eGFP*, generating *pMSCV-Pax7DN-IRES-eGFP* (Pax7DN RV) and *pMSCV-Pax3DN-IRES-eGFP* (Pax3DN RV) respectively, used to repress Pax7 or Pax3 transcriptional targets. Retroviral constructs, together with an ecotropic packaging plasmid, were transiently co-transfected into 293T cells to produce non-replicating retrovirus and the supernatant harvested.

### Retroviral infection

A total of 1,000 C2C12 cells, 2,000 NIH 3T3cells or 5,000 primary myoblasts were plated in each well of LAB-TEK® 8-well chamber slides (Nunc). After 48 h, the medium was replaced with a 1∶1 dilution of 293T retroviral supernatant with 4 µg/ml polybrene and incubated at 37°C for 3 h, before the cells were rinsed and placed in fresh medium. For the studies on the effects of constitutive Pax construct expression on differentiation, myogenic cells were left to reach confluency, at which point they began to spontaneously differentiate and fuse into myotubes, and were analysed five days post-infection. To infect satellite cells associated with myofibres, they were exposed to a 1∶10 dilution of the supernatant after 24 hours in culture, and the cells were then analysed 48 hours later. Where used, BrdU was added to the medium at a final concentration of 10 µM for 2 h prior to fixation.

### Clonal proliferation assay

Retroviral infection of C2C12 and NIH 3T3 cells was carried out in 6-well plates, and cells were then cultured for a further 24 h before trypsinisation and re-plating at clonal density (50 cells/cm^2^) in LAB-TEK® 2-well chamber slides (Nunc). Retroviral infection of satellite cells was carried out in myofibre cultures 24 h after plating. Myofibres were removed 24 h after infection, and the migrating satellite cell-derived myoblasts were trypsinised and re-plated at clonal density in chamber slides. After a further 72 h in culture, cells were fixed, immunostained and the numbers of cells in distinct eGFP-expressing colonies were counted.

### Immunocytochemistry

Cell cultures or myofibres were fixed in 4% paraformaldehyde/PBS for 10 minutes, permeabilised with 0.5% (v/v) Triton X-100 in PBS and then blocked using 10% (v/v) goat serum and 10% (v/v) swine serum in PBS. Primary antibodies used were monoclonal rat anti-BrdU clone BU1/75 (Abcam), monoclonal mouse anti-myogenin clone F5D (Developmental Studies Hybridoma Bank), polyclonal rabbit anti-myogenin (Santa Cruz), monoclonal mouse anti-MyoD clone 5.8A (DakoCytomation), polyclonal rabbit anti-MyoD (Santa Cruz), monoclonal mouse anti-Pax7 (Developmental Studies Hybridoma Bank), monoclonal mouse anti-Pax3 (Developmental Studies Hybridoma Bank) polyclonal rabbit anti-GFP (Invitrogen) and monoclonal mouse anti-MyHC (MF20) (Developmental Studies Hybridoma Bank). Primary antibodies were visualised with species-specific or isotype-specific fluorochrome-conjugated secondary antibodies (Invitrogen) before mounting in Faramount fluorescent mounting medium (DakoCytomation) containing 100 ng/ml 4,6-diamidino-2-phenylindole (DAPI).

### Western Blotting

Lysates were collected from C2C12 cells either 72 h or 1 week post-infection in LDS sample buffer (Expedeon) supplemented with 20 mM dithiothrietol. Lysates were separated by 4–20% gradient sodium dodecyl sulphate polyacrylamide gel electrophoresis (RunBlue: Expedeon) and blotted onto nitrocellulose membranes using the iBlot system (Invitrogen). Membranes were blocked in 2% non-fat milk PBS with 0.5% Tween-20 and incubated with the following primary antibodies overnight at 4°C: monoclonal mouse anti-Pax7 (1∶10); monoclonal mouse anti-MyoD clone 5.8A (1∶100) or polyclonal rabbit anti-GFP (1∶200), then washed and incubated with species-matched Horseradish Peroxidase-conjugated secondary IgG. Protein bands were visualised using enhanced chemiluminescence (GE Healthcare). All blots were stripped with Restore Stripping Buffer (Thermo Scientific) and re-probed with monoclonal mouse anti-β-tubulin clone E7-c (Developmental Studies Hybridoma Bank: 1∶1000) as a loading control.

### Quantitative RT-PCR

Total RNA was isolated using an RNeasy kit (Qiagen) from infected C2C12 cells 72 h post-infection, and cDNA prepared using the Quanti-Tect kit (Qiagen). QPCR was performed on an Mx3005P QPCR system (Stratagene) using Brilliant II SYBR green reagents with ROX reference dye (Stratagene) with the following primers, designed using Primer-BLAST (NCBI): *Myf5* (F – 5′ TGAGGGAACAGGTGGAGAAC 3′, R – 5′ AGCTGGACACGGAGCTTTTA 3′); *MyoD* (F – 5′ AGCACTACAGTGGCGACTCA 3′, R - 5′ GCTCCACTATGCTGGACAGG 3′); *myogenin* (F – 5′ CTACAGGCCTTGCTCAGCTC 3′, R – 5′ AGATTGTGGGCGTCTGTAGG 3′) and *Gapdh* (F – 5′ GTGAAGGTCGGTGTGAACG 3′, R – 5′ ATTTGATGTTAGTGGGGTCTCG 3′). Expression levels of *Myf5*, *MyoD* and *myogenin* were normalised to expression levels of *Gapdh* control gene and are represented as fold change over values derived from control RV-infected cultures. Results show duplicate QPCR analysis from two independent experiments (*n* = 2+2). Statistical analysis was performed using Relative Expression Software Tool (REST 2008).

Semi-quantitative RT-PCR analysis for *Pax3* and *Pax7* was conducted using *Taq* polymerase with primers for *Pax3* (F – 5′ GGGAACTGGAGGCATGTTTA 3′, R - 5′ GTTTTCCGTCCCAGCAATTA 3′) and primers for Pax7 that amplify both the endogenous and dominant-negative mRNA, *Pax7* (F 5′ – CCGTGTTTCTCATGGTTGTG 3′, R – 5′ GAGCACTCGGCTAATCGAAC 3′).

### Image capture and quantitative analysis

Images were captured on a Leica DMR microscope using Metamorph Image analysis software. Cell size was quantified using SigmaScan Pro. Myotube size was evaluated by measuring a sequence of minor axes perpendicular to the major axis of each distinguishable myotube. The average minor axis was calculated as the mean of a series of axis values taken throughout the length of each myotube. Images were optimized globally and assembled into figures using Adobe Photoshop CS3.

## Results

The role of Pax3 in myogenesis has mainly been examined during embryonic development [e.g. 27]. Here we wished to examine the effects of Pax3 on myogenesis in adult, and used both primary satellite cells [10] and the C2C12 myogenic cell line, derived from regenerating adult mouse skeletal muscle [28].

Pax7 is expressed in satellite cells in muscles from throughout the body [10], [16], while the *Pax3* locus is active in satellite cells in a subset of muscles, including many in the forelimb [11], [15]. We were unable to detect endogenous Pax3 protein in any satellite cell on freshly isolated myofibres from any muscles examined (including from the forelimb), using a monoclonal mouse anti-Pax3 antibody (obtained from DSHB) (data not shown). We also examined Pax3 protein in proliferating C2C12 myoblasts, but were unable to detect endogenous Pax3 protein at any stage, including in reserve cells (quiescent-like cells, opting out of immediate differentiation [29]) (data not shown).

**Figure 1:**
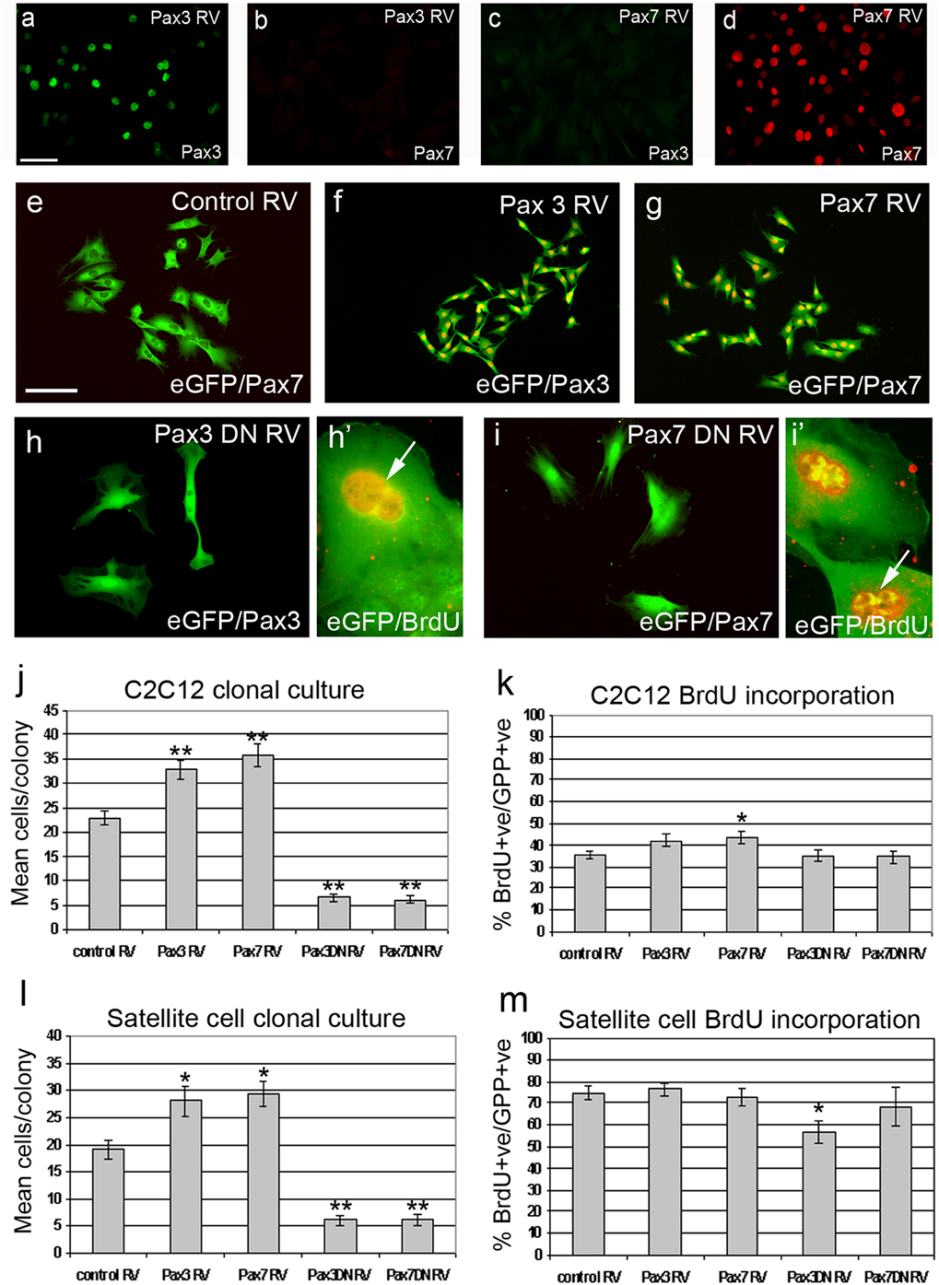
Pax3 and Pax7 regulate the rate of myogenic cell division. Pax3 RV and Pax7 RV were used to infect a subclone of C2C12 with low endogenous Pax7 levels, to first determine if Pax3 or Pax7 could regulate each other. Pax3 RV-infected cells were only recognized by the anti-Pax3 monoclonal antibody (a) and not by the anti-Pax7 antibody (b), whereas Pax7 RV-infected cells were recognized only by the anti-Pax7 antibody (d) and not by the anti-Pax3 (c). To determine how Pax3 or Pax7 affects myogenic cell proliferation, C2C12 and satellite cell-derived myoblasts were infected with control RV, Pax3 RV, Pax7 RV, Pax3DN and Pax7DN, plated at clonal density, cultured and fixed 72 h later. They were then co-immunostained for either eGFP (green) and Pax3 (red), or eGFP (green) and Pax7 (red), and counterstained with DAPI to identify all nuclei. All cells present in a clone are shown (e–i). Pax3 and Pax7 RV-infected C2C12 (f and g) and satellite cell-derived myoblast clones produced more cells per colony compared to control (quantified in j and l). In contrast, constitutive expression of Pax3DN or Pax7DN generated C2C12 clones (h and i) with significantly less cells (quantified in j) with similar results obtained with satellite cell-derived myoblasts (quantified in l). Despite the different proliferation rates, constitutive expression of Pax3, Pax7 or their dominant-negative versions did not generally alter the percentage of C2C12 or plated satellite cells incorporating BrdU 72 h post-infection (quantified in k and m). Interestingly, BrdU labelling (BrdU+ve) of eGFP expressing cells (eGFP+ve) infected with Pax3DN (h′) or Pax7DN (i′) revealed that many cells had two nuclei (arrowed). Scale bar represents 40 µm (except h′ and i′). Values are population means±SEM of 15–20 clones from each of 3 independent experiments, where an asterisk denotes significant difference at *p*<0.05, while two asterisks denotes significant difference at *p*<0.0001, from controls using Mann-Whitney.

To both investigate cross-regulation between *Pax* genes in myogenic cells, and confirm the specificity of the anti-Pax3 antibody for Pax3, we infected low-density cultures of a C2C12 subclone (with low endogenous Pax7 levels) with Pax3 RV or Pax7 RV, which resulted in co-expression of Pax3, or Pax7 respectively, together with eGFP from the *IRES-eGFP* in the retroviral backbone (Figure 1a–d). Pax3 IgG2α monoclonal antibody bound only to the nuclei of cells infected with Pax3 RV, but not those infected with Pax7 RV, while the Pax7 IgG1 monoclonal antibody bound only to the nuclei of cells infected with Pax7 RV and not Pax3 RV (Figure 1a–d). Therefore, expression of either *Pax* gene did not induce expression of its paralogue, and we confirm that the Pax3 and Pax7 antibodies each have high specificity for their target protein (Figure 1a–d).

### Pax3 and Pax7 regulate the rate of cell division in myogenic cells

It was noted that bulk cultures infected with Pax3 RV or Pax7 RV appeared to have more cells than those infected with control RV. To further investigate a potential role for Pax3 and Pax7 in the control of proliferation rate, C2C12 cells and primary satellite cell-derived myoblasts were infected with RV and then trypsinised and re-plated at clonal density. After a further 72 h in culture, cells were fixed and immunostained and the number of cells per each eGFP-expressing clone counted. Mean cell number in clones derived from cells infected with control RV was not significantly different to that in clones derived from non-infected cells, showing that although RV are only integrated into actively-dividing cells, infected cells are a representative sample of the whole proliferation-competent population. Clones derived from Pax3 RV- or Pax7 RV-infected C2C12 cells (Figure 1e–g, quantified in j) and satellite cells (Figure 1l) contained significantly greater numbers of progeny than clones derived from cells expressing control RV (*p*<0.001 quantified in j and l).

In addition to using constitutive expression of Pax3 and Pax7 to explore the role of these genes, we also used dominant-negative versions [15], [30]. In the Pax3DN and Pax7DN fusion proteins, the *Pax* gene sequences directing DNA binding (and so target gene selection) are fused in frame with the active repression domain of *Drosophila* Engrailed, so repress Pax3 or Pax7 transcriptional targets. Infection with Pax3DN RV or Pax7DN RV dominant-negative constructs was identified by production of eGFP from *IRES-eGFP* in the viral backbone. eGFP+ve infected cells were not recognized by the Pax3 or Pax7 antibodies, since the relevant epitopes are not included in the dominant-negative constructs. Infection of myoblasts with either Pax3DN RV or Pax7DN RV dominant-negative constructs resulted in a significant reduction in the mean number of cells per colony in both C2C12 (Figure 1h and i, *p<0.001* quantified in j) and satellite cell-derived myoblasts (*p<0.001* - Figure 1l). Infected cells attached to the substrate, and did not have an apoptotic appearance.

C2C12 cells and primary satellite cells were infected with control RV, Pax3 RV, Pax7 RV, Pax3DN RV or Pax7DN RV, and then treated with a pulse of BrdU for 2 h immediately before fixation. When compared with cells infected with the control RV, infection of cells with any of the other four vectors did not result in a significant difference between the proportions of cells that were actively synthesising DNA (Figure 1k and m). Thus, activation or inhibition of Pax3/Pax7 transcriptional targets does not cause dividing cells to cease DNA synthesis, or greatly affect the length of S-phase.

### Pax3 and Pax7 can regulate cell size and morphology of myogenic cells

**Figure 2:**
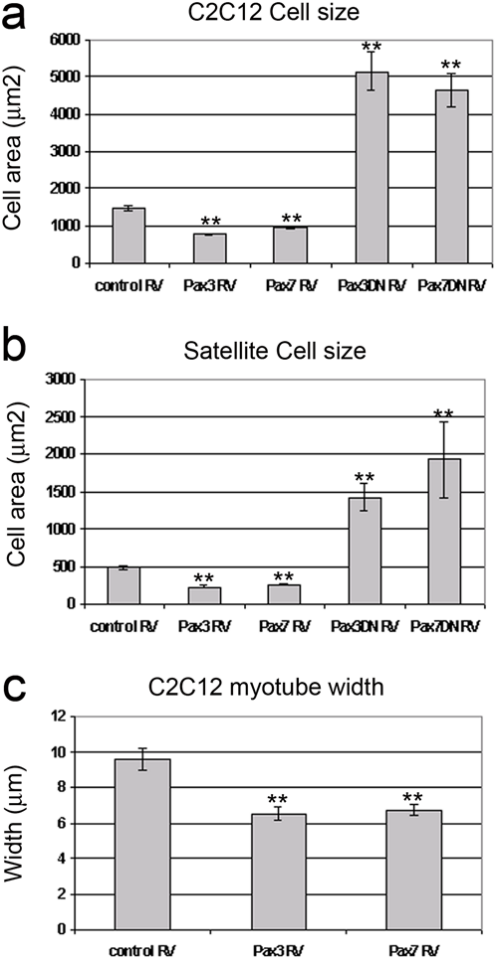
Pax3 and Pax7 can regulate the cell size of myogenic cells. To examine how altered Pax gene function affected cell size, C2C12 and satellite cell-derived myoblasts were infected with retroviral constructs encoding Pax3, Pax7, Pax3DN or Pax7DN, together with empty vector serving as control. Infected cells were plated at clonal density, fixed after 72 h, immunostained, counterstained with DAPI and cell size determined using SigmaScan Pro image analysis software. For both C2C12 (a) and satellite cell-derived myoblasts (b), constitutive Pax3 or Pax7 expression resulted in significantly smaller cells, while the presence of Pax3DN or Pax7DN caused the cells to get bigger. Indeed, even the myotubes formed from Pax3 RV- or Pax7 RV-infected C2C12 myoblasts were significantly smaller than controls (c). Values are population means±SEM of 17–60 cells/myotubes in total from 3 independent experiments, where an asterisk denotes significant difference (*p*<0.0001) from controls using Mann Whitney.

A surprising and unexpected adjunct effect of constitutive expression of Pax3 or Pax7, or dominant negative inhibition of their transcriptional targets, was a rapid and pronounced change in cell size and morphology. Constitutive expression of Pax3 or Pax7 resulted in smaller, rounder cells, an effect that was most pronounced in C2C12 cells when compared to actively dividing control cells (Figure 1e–g). Conversely, dominant-negative inhibition of Pax3/Pax7 transcriptional targets resulted in a dramatic increase in cell size coupled with flatter, more irregular morphology and frequent doubling of nuclei (Figure 1h′ and i′). Cell size was quantified using SigmaScan Pro image analysis software, and revealed that in comparison to cells infected with the control vector, C2C12 cells (Figure 2a) or satellite cells (Figure 2b) infected with Pax3 RV or Pax7 RV were significantly smaller, while cells infected with dominant negative Pax3DN RV or Pax7DN RV were significantly larger (*p*<0.0001).

Thus the increased rate of division in myogenic cells constitutively expressing Pax3 or Pax7 was coupled with a decrease in cell size. Notably however, neither dominant-negative vector completely inhibited cell division, and the proportion of cells actively synthesising DNA was relatively unchanged between the different groups.

### Pax3 stimulates MyoD expression and delays myogenic differentiation

Exposure of satellite cells to the mitogen-rich tissue culture environment results in rapid activation of quiescent satellite cells to co-express MyoD with Pax7 in 95–100% of cells [10]. In low-density C2C12 cultures, only ∼50% of proliferating cells express MyoD at any given time. We were therefore able to use satellite cells and C2C12 cells as tools to investigate the effects of constitutive expression of Pax3, or dominant negative inhibition of the transcriptional targets of Pax3 and Pax7, in cells with differing background levels of myogenic transcription factor expression.

**Figure 3:**
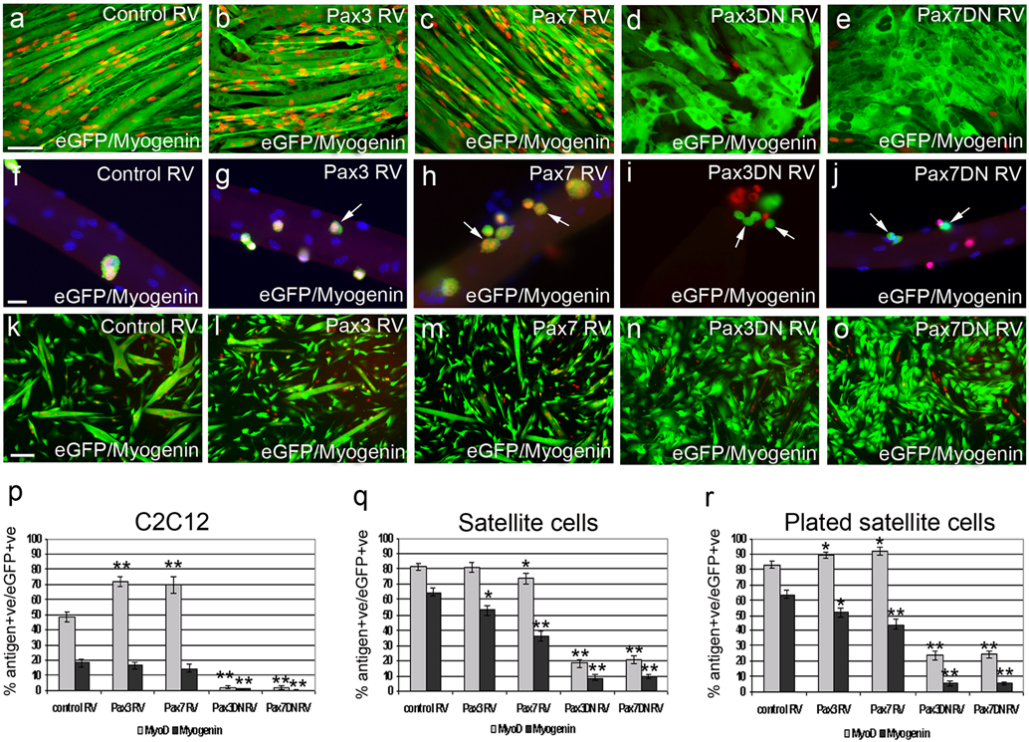
Pax3/Pax7 transcriptional activity is required for myogenin expression. To test the effects of perturbing Pax3 and Pax7 function on myogenicity, cells were infected with retroviral constructs encoding Pax3, Pax7, Pax3DN or Pax7DN, together with empty vector serving as control. Cells underwent further culture and were then co-immunostained for either eGFP (green) and MyoD (red), or eGFP (green) and myogenin (red). C2C12 cells infected with control RV (a), Pax3 RV (b), Pax7 RV (c), Pax3DN RV (d) and Pax7DN RV (e) and co-immunostained for eGFP and myogenin 120 h after infection. Constitutive Pax3 or Pax7 expression resulted in more infected cells (eGFP+ve) with MyoD, while constitutive Pax3DN or Pax7DN caused less eGFP+ve cells to co-express MyoD and myogenin (quantified in p after 72 h). Satellite cells were infected with control RV (f), Pax3 RV (g), Pax7 RV (h), Pax3DN RV (i) and Pax7DN RV (j) while retained in their niche on the myofibre and cultured before co-immunostaining for eGFP (green) and myogenin (red). The presence of constitutively expressed Pax3 (g) or Pax7 (h) was compatible with myogenin expression (arrows), but there were reduced numbers of infected eGFP+ve satellite cell progeny containing myogenin (quantified in q). Many infected GFP+ve cells expressing Pax3DN (i - green - arrow) and Pax7DN (j - green - arrow) did not contain myogenin (i and j - red) (quantified in q). Similar results were obtained when plated satellite cell-derived myoblasts were infected with control RV (k), Pax3 RV (l), Pax7 RV (m), Pax3DN RV (n) and Pax7DN RV (o) and co-stained for eGFP (green) and myogenin (red), 72 h post infection (quantified in r). Counterstaining with DAPI was used to identify all nuclei present. Scale bar for each row equals 40 µm, except for k–o where it represents 100 µm. Values are population means±SEM of at least 3 independent experiments, where an asterisk denotes significant difference at *p*<0.05, while two asterisks denotes significant difference at *p*<0.0001, from controls using Mann-Whitney.

Constitutive expression of Pax3 under low-density culture conditions led to a significant increase in the number of MyoD-expressing C2C12 cells (Figure 3p). Pax7 RV infections were included as controls for Pax3 and Pax7DN RV and gave results consistent with our previous observations [21]. The proportion of C2C12 myoblasts expressing myogenin remained unchanged compared to control-infected cells (Figure 3a–c, quantified in p). Infection of C2C12 cells with either Pax3DN RV or Pax7DN RV dominant-negative constructs led to a rapid (within 24 h) and significant (*p*<0.0001) reduction in the proportion of cells expressing either MyoD or myogenin protein (Figure 3d and e, quantified in p), suggesting that although all C2C12 cells lack Pax3, Pax transcriptional targets are normally activated.

While retroviral-mediated constitutive expression of Pax3 did not result in any change in MyoD expression in satellite cells retained in their niche on an isolated myofibre (Figure 3q), significantly fewer started to express myogenin after 72 h in culture (Figure 3f–h, quantified in q). These observations are in accordance with our previous findings on constitutive Pax7 expression [21]. As with C2C12 cells, infection of satellite cells with Pax3DN RV or Pax7DN RV led to significant reductions (*p*<0.0001) in the number of cells expressing either MyoD or myogenin (Figure 3i and j, quantified in q). Therefore the presence of constitutively expressed Pax3 in satellite cells can reduce the proportion of cells that initiate expression of myogenin, and inhibition of Pax3/Pax7 function results in almost complete abrogation of myogenin expression. Plated satellite cell-derived myoblasts also exhibited an increase in the number of cells containing MyoD in the presence of constitutive Pax3 or Pax7 (Figure 3r), while showing significant inhibition of myogenin induction in the presence of Pax3, Pax7, Pax3DN and Pax7 DN, compared to control infected cultures (Figure 3k–o, quantified in r).

### Inhibition of Pax3/Pax7 transcriptional targets prevents myogenic differentiation

**Figure 4:**
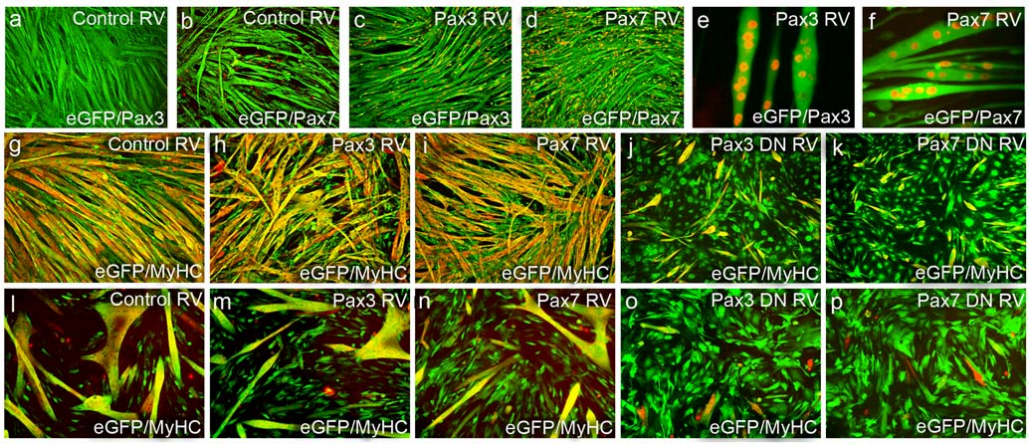
Inhibition of Pax3/Pax7 transcriptional targets prevents myogenic differentiation. To test the effects of perturbing Pax3 and Pax7 function on the ability of myoblasts to fuse into multinucleated myotubes, C2C12 and plated satellite cell-derived myoblasts were infected with retroviral constructs encoding Pax3, Pax7, Pax3DN or Pax7DN, together with empty vector serving as control. Cells were then allowed to differentiate and fuse for 5 days and immunostained. Control RV-infected C2C12 co-immunostained for either eGFP (green) and Pax3 (red) or eGFP (green) and Pax7 (red) showed that Pax3 was not expressed in reserve cells (a) while Pax7 was (b), with neither protein present in myotubes. C2C12 cells still efficiently fused into myotubes in the presence of constitutively expressed Pax3 (c) and Pax7 (d), with ectopic, retroviral-driven expression of Pax3 (e) and Pax7 (f) in the nuclei of multinucleated satellite cell-derived myotubes. Infection of C2C12 cells with control RV (g), Pax3 RV (h), Pax7 RV (i), Pax3DN RV (j) and Pax7DN RV (k) and co-immunostaining for eGFP (green) and myosin heavy chain (MyHC-red) confirmed that constitutive Pax3 (h) and Pax7 (i) expression did not perturb myotube formation, in contrast to Pax3DN (j) and Pax7DN (k), which effectively prevented it, with very few myotubes containing both eGFP and MyHC (yellow). Similar results were obtained when plated satellite cell-derived myoblasts were infected with control RV (l), Pax3 RV (m), Pax7 RV (n), Pax3DN RV (o) and Pax7DN RV (p) and co-immunostained for eGFP (green) and MyHC (red). Counterstaining with DAPI was used to identify all nuclei present. All experiments were repeated at least 3 times. Scale bar represents 40 µm.

Both the presence of constitutively expressed Pax3 or Pax7, and inhibition of Pax3 and Pax7 transcriptional targets using the dominant-negative constructs, delay the induction of myogenin. We next asked whether this delay was temporary, or if myogenic differentiation was inhibited. Differentiation and fusion of myogenic cells into myotubes in vitro can be induced by allowing cultures to reach confluence. As expected, C2C12 cells or primary satellite cell-derived myoblasts infected with control RV and cultured to confluence (around 120 h under our experimental conditions) readily formed large multinucleate myotubes that expressed Myosin Heavy Chain (MyHC), a marker of sarcomere assembly during terminal differentiation (Figure 4). Reserve cells arising in post-differentiated cultures of both C2C12 (Figure 4b) and plated satellite cell-derived myoblasts were positive for Pax7. While Pax7 was robustly expressed by reserve cells, we could find no evidence of the presence of Pax3 protein (Figure 4a). Infection of C2C12 cells (Figure 4c and d) or satellite cell-derived myoblasts (Figure 4e and f) with Pax3 RV or Pax7 RV did not prevent the development of multinucleate myotubes, though in both cases there was a slight delay (∼24 h) in fusion. Immunostaining for Pax3 or Pax7 confirmed the presence of each protein in many nuclei of myotubes derived from RV-infected C2C12 and satellite cell-derived myoblasts (Figure 4c–f) which did not perturb sarcomere assembly, as shown by the presence of MyHC (Figure 4g–i and l–n). However, myotubes formed from Pax3 RV- or Pax7 RV-containing C2C12 cells were significantly thinner than those formed from control RV-infected C2C12 cells, as determined using measurements taken from the minor axis of individual myotubes (Figure 2c).

By contrast, infection with Pax3DN RV or Pax7DN RV dominant-negative constructs blocked differentiation, such that no myotubes were formed and MyHC was rarely expressed in both C2C12 (Figure 4j and k) and primary satellite cell-derived cultures (Figure 4o and p). Therefore, although endogenous expression of Pax3 and Pax7 is restricted to undifferentiated myogenic cells, neither factor has a major inhibitory effect on myogenic differentiation. Indeed, activation of Pax3 and Pax7 DNA targets is necessary for the differentiation program to proceed.

### Effects of constitutive Pax3 and Pax7 on *Myf5*, *MyoD* and *myogenin* mRNA

**Figure 5:**
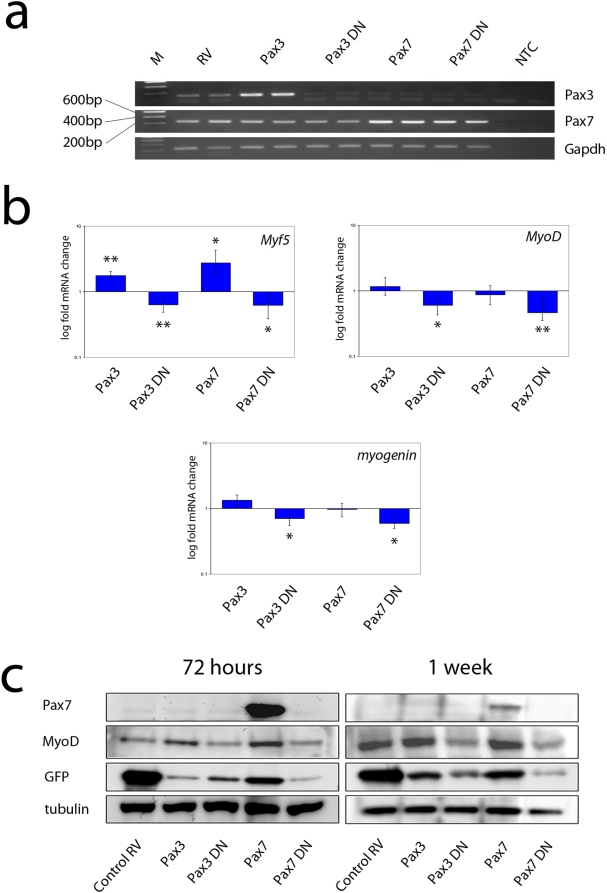
Quantitative analysis of the effects of Pax3 and Pax7 on myogenic gene expression. C2C12 were infected with control RV, Pax3 RV, Pax7 RV, Pax3DN RV or Pax7DN RV and analysed for mRNA (72 h later) and protein levels either 72 h or 1 week later. Semi-quantitative RT-PCR showed that constitutive Pax7 expression did not induce endogenous *Pax3* mRNA expression, while Pax3DN did not down-regulate endogenous *Pax7* mRNA (a – M: DNA ladder; NTC: no template control; amplicon sizes – *Pax3*: 151 bp; *Pax7*: 247 bp; *Gapdh*: 250 bp, representative experiments shown in duplicate). QPCR analysis of *Myf5*, *MyoD* and *myogenin* expression levels 72 h post-infection demonstrated that constitutive Pax3 or Pax7 expression both increased *Myf5* levels, whereas the dominant-negative versions had the opposite effects and decreased *Myf5* expression (b). Pax3DN or Pax7DN also suppressed both MyoD and myogenin expression (b). Data show mean fold change from control RV-infected cells (mean±SEM, from duplicate measurements from 2 independent experiments *n* = 2+2). Hypothesis testing performed using REST analysis where an asterisk denotes significant difference at *p*<0.05, while two asterisks denotes significant difference at *p*<0.01. Western blot analysis of infected cells showed that constitutive Pax3 or Pax7 expression increased MyoD protein levels, while the dominant-negative versions both resulted in less MyoD protein being present (c). Western blotting demonstrated that although the infection rates were similar, each construct produced eGFP with differing efficiencies from the *IRES-eGFP* in the retroviral backbone.

In order to better understand the dynamics of myogenic gene expression in response to constitutively expressed Pax3 and Pax7, we also examined the mRNA levels. RNA was isolated from C2C12 cells that had been infected with control RV, Pax3 RV, Pax7 RV, Pax3DN RV or Pax7DN RV, and then cultured for 72 h. The results of semi-quantitative RT-PCR for *Pax3* and *Pax7* transcripts confirmed our immunocytochemical data. *Pax3* mRNA was only present at high levels in cultures infected with the Pax3 RV, whereas *Pax7* mRNA was detected in all cultures, but was present at much higher levels in cultures infected with Pax7 RV (Figure 5a). The Pax7 primers also recognised transcript produced from the Pax7DN construct, but Western blotting using the Pax7 antibody confirmed that only cultures infected with Pax7 RV contained high levels of Pax7 protein (Figure 5c). We could not assess Pax3 protein levels by Western blot due to the unsuitability of the antibody for this application.

We next used QPCR to compare the relative expression levels of the myogenic regulatory factors *Myf5*, *MyoD* and *myogenin*. Constitutive expression of either Pax3 or Pax7 resulted in a significant increase in *Myf5* mRNA levels, whereas expression of either Pax3DN or Pax7DN resulted in a decrease in *Myf5* expression (Figure 5b). We could not assess levels of Myf5 protein due to the absence of an appropriate antibody. Expression of Pax3DN or Pax7DN also resulted in significantly reduced levels of *MyoD* and *myogenin* mRNAs (Figure 5b), consistent with immunostaining results (Figure 3). Interestingly, whereas our earlier immunocytochemical data showed that constitutive expression of Pax3 or Pax7 led to an increased proportion of MyoD-expressing cells, our QPCR data did not show an increase in *MyoD* mRNA (Figure 5b). Western blotting of cell lysates confirmed that infection of cells with Pax3 or Pax7 RV resulted in an increase in MyoD protein, whereas infection with retroviruses encoding Pax3DN or Pax7DN resulted in a decrease in MyoD protein (Figure 5c). These data suggest that whereas *Myf5* and *myogenin* are regulated by Pax proteins at the gene expression level, MyoD is regulated by a different mechanism, perhaps, for instance, at the level of protein stability.

### Pax3/Pax7 transcriptional targets regulate proliferation and cell size independently of the myogenic program

We have here provided evidence that Pax3 and Pax7 can influence the rate of cell division in myogenic cells, and also shown that these factors can regulate the levels of Myf5 and MyoD: myogenic regulatory factors normally associated with activated and proliferating satellite cells. To investigate whether the effects on cell division and size were related to the myogenic program, we also used our Pax-encoding retroviruses to infect NIH 3T3 mouse fibroblasts.

**Figure 6:**
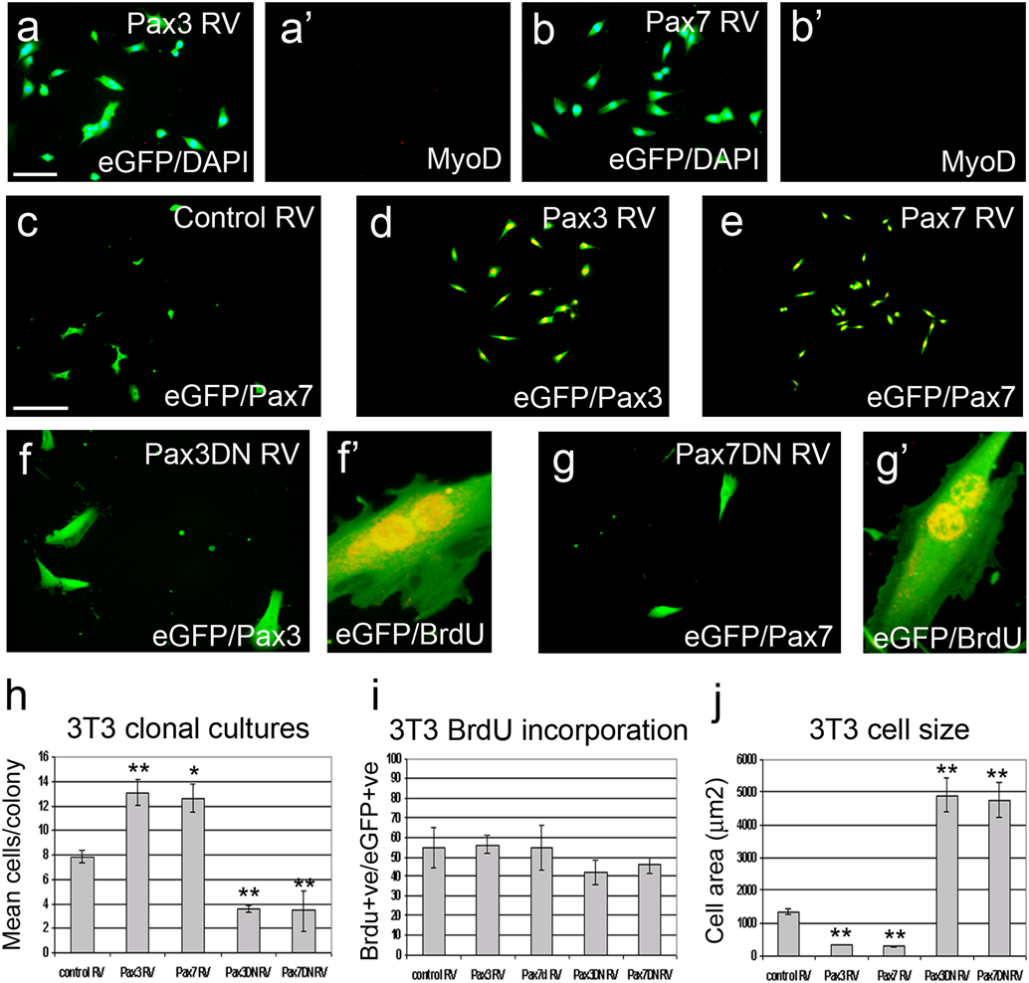
Pax3/Pax7 transcriptional targets can regulate proliferation and cell size independently of the myogenic program. To establish whether the effects on cell proliferation and size were a result of interactions of Pax proteins with myogenic genes, NIH 3T3 fibroblasts were also infected with either Pax3 RV (a and d), Pax7 RV (b and e), control RV (c), Pax3DN RV (f) or Pax7DN RV (g). Infected cells were plated at clonal density and analysed 72 h post-infection by co-immunostaining for either eGFP (green) and MyoD (red); eGFP (green) and Pax3 (red); eGFP (green) and Pax7 (red) or eGFP (green) and BrdU (red), before counterstaining with DAPI. NIH 3T3 fibroblasts did not express Pax3 or Pax7 (c) and constitutive expression of these proteins (a and b) did not elicit the initiation of the myogenic program, as shown by their failure to induce MyoD (a′-b′). As with myogenic cells, constitutive Pax3 (d) and Pax7 (e) expression resulted in larger clone sizes, while Pax3DN (f) and Pax7DN (g) had the opposite effect, and significantly reduced clone size (quantified in h). Infection of NIH 3T3 cells with Pax3DN RV (f′) or Pax7DN RV (g′) followed by a 2 h BrdU pulse after 72 h, indicated that S-phase length was not drastically altered (percentage of infected cells containing BrdU (BrdU+ve/eGFP+ve) quantified in i). Cells with double nuclei were present (f′ and g′). Again, as with myogenic cells, constitutive Pax3 and Pax7 also caused NIH 3T3 cells to become significantly smaller, while the presence of the dominant-negative versions resulted in an increase in mean cell size using SigmaScan Pro (quantified in j). Scale bar equals 40 µm (except d′ and e′). Values are population means±SEM of 6–20 clones from each of 3 independent experiments, where an asterisk denotes significant difference at *p*<0.05, while two asterisks denotes significant difference at *p*<0.0001, from controls using Mann-Whitney.

NIH 3T3 cells infected with the control RV did not contain immunodetectable levels of Pax3 or Pax7 (Figure 6c) and infection with Pax3 RV or Pax7 RV did not result in myogenic conversion, as assessed by the absence of MyoD (Figure 6a–b′), myogenin and MyHC proteins, and a failure to form myotubes (data not shown). However, we observed effects on cell division and cell size that were similar to those seen in myogenic cells. When plated at clonal density, NIH 3T3 cells expressing Pax3 or Pax7 generated colonies that contained significantly larger numbers of progeny than colonies derived from cells infected with control RV (Figure 6c–e, quantified in h). Conversely, NIH 3T3 cells expressing either Pax3DN or Pax7DN generated colonies that contained significantly fewer progeny than colonies derived from cells infected with control RV (Figure 6f–g, quantified in h). The proportion of cells containing BrdU after a 2 h pulse was not significantly different between conditions (Figure 6i). As with myogenic cells, many cells expressing the dominant-negative constructs exhibited doubling of nuclei (Figure 6f′ and g′). Moreover, when compared with cells infected with control RV, constitutive expression of Pax3 or Pax7 caused significant decreases in the mean cell size of NIH 3T3 cells, while dominant-negative inhibition of Pax3 or Pax7 transcriptional targets caused significant increases in cell size (Figure 6c–g′, quantified in j). Thus, Pax3 and Pax7 can regulate the rate of cell division and cell size independently of MyoD and of other factors specific to myogenic cells.

## Discussion

*Pax* gene function has been widely examined in mouse models and the roles of Pax3 and Pax7 during developmental myogenesis are relatively well understood [1]. However, mice null for *Pax3* die in utero [31], while loss of *Pax7* severely compromises postnatal muscle development and growth [15]–[17]. Mouse models in which *Pax3* alleles have been targeted with *Pax3DN* [3] or the stronger transcriptional activator, *Pax3-FOXO1A* [32], die shortly after birth. Furthermore, *Pax3* and *Pax7* are also expressed extensively in neural tissue, and Pax mutant mice exhibit perturbed neural function, which could potentially have effects on satellite cell development or function. Satellite cells formed in Pax7-null mice are rapidly depleted from muscle following birth [15]–[17], but it is unknown whether this occurs as a result of defects acquired during development, or through a direct requirement for Pax7 postnatally. To study the role of *Pax* genes in normal adult satellite cell function, we therefore used primary satellite cells derived from wild type mice, in combination with the satellite cell-derived C2 myogenic cell line [28], where necessary.

While Pax7 is expressed by most, if not all, freshly isolated satellite cells [10], [16], we were unable to detect Pax3 protein in any. This included muscles in which the *Pax3* locus has been shown to be active, such as those of the forelimb and diaphragm [11], [14], [15]. It has been shown that Pax3 is transiently detectable during satellite cell activation, with a possible role in the expansion of the population mooted [11]–[13]. Our observations that constitutive expression of Pax3 or Pax7 causes both primary satellite cell-derived myoblasts and C2C12 immortalised myoblasts to increase their rate of cell division, suggests that indeed, Pax7 and Pax3 function to promote expansion of the adult muscle precursor cell pool, consistent with the roles of these genes during embryonic development [e.g. 2].

The ability of Pax3 to activate the myogenic program in various embryonic tissues, including neural cells, and in the pluripotent P19 stem cell line, is well documented [e.g. 27], [30]. However, in adult-derived cells, there are conflicting reports of the effects of Pax3, with both an inhibition of myogenic differentiation [22] or no effects [24], having been recorded. Our observation that constitutive expression of Pax3 is compatible with differentiation of primary satellite cells is consistent with a recent report documenting efficient differentiation of primary myoblasts after transfection with a *Pax3* vector [24] and our previous observations on Pax7 [21]. Interestingly, alveolar rhabdomyosarcoma is often associated with a chromosomal translocation that creates a chimeric protein, PAX3-FOXO1A [33]. Target gene selection in PAX3-FOXO1A is directed by the *Pax3* sequences and expression of this strong transcriptional activator in fibroblasts can directly promote myogenin transcription and activate a myogenic program [34], to the extent of conversion into fully differentiated myotubes [35]. However, since both Pax3 and Pax7 can delay the induction of myogenin expression, and so differentiation, it is possible that where inhibitory effects of Pax3 on myogenic differentiation have been reported, the time of analyses might have fallen within this period of pronounced delay [22], [23]. Whilst in our experiments, constitutive expression of Pax3 or Pax7 did not prevent expression of MyHC or fusion into syncytia, the resultant myotubes had a significantly smaller diameter, showing that ectopic Pax3 or Pax7 restricts myoblast accretion and/or myotube growth. Our results indicate that the inhibitory effect of Pax3 or Pax7 on myogenic differentiation may result from an extension of the normal proliferative phase.

It has been previously reported that dominant-negative inhibition of Pax3 in P19 pluripotent stem cells prevents the adoption of the myogenic, but not cardiac, skeletal lineage [30], demonstrating that dominant-negative constructs can be a useful tool for examining lineage-specific transcriptional regulation. In plated myogenic cells from juvenile (3-wk) mice, adenoviral delivery of Pax3DN or Pax7DN inhibited MyoD but not Myf5, and did not prevent myogenin induction, although the later stages of differentiation were not examined [15]. In our experimental system using defined adult satellite cell-derived myoblasts, or myogenic cell lines, dominant-negative inhibition of either Pax3 or Pax7 transcriptional targets resulted in essentially indistinguishable cell phenotypes: myogenic cells down-regulated *Myf5* mRNA and both *MyoD* mRNA and protein, and failed to induce *myogenin* mRNA or protein and fuse into myotubes.

Dominant-negative constructs also slowed the rate of proliferation and induced a dramatic increase in cell size. DNA synthesis (measured by BrdU uptake) was maintained, showing that the cells were still viable and undergoing cell division. Interestingly, we observed frequent doubling of nuclei, implying a failure or retardation of cleavage. This behaviour is analogous to that previously reported in cells treated with very low doses of the cleavage inhibitor cytochalasin. Whereas cells treated with high doses of cytochalasin become binucleate and arrest in G1, cells treated with very low doses become binucleate but maintain DNA synthesis and eventually divide [36]. The inhibition of myogenic differentiation and severe retardation of proliferation that resulted from dominant-negative inhibition of Pax3/Pax7 transcriptional targets is not a phenomenon specific to transformed cell lines because we observed the same effects in primary satellite cell-derived myoblasts. Similarly, primary myoblasts isolated from *Pax7* null mice also display perturbation of the cell cycle and consequently, decreased colony size [15], [17]. It is possible that the altered cell morphology caused by the presence of PaxDN constructs could be attributable to perturbed expression of cell surface antigens, since Pax3 is known to affect cell surface properties [37], which may affect cell shape by changed adherence to the tissue culture substrate.

The paired-domain and homeodomain of Pax3 and Pax7 are responsible for directing DNA binding, and are highly conserved between the two proteins. Mouse Pax3 and Pax7 paired domains differ by 8/128 amino acids, and their homeodomains by only 2/60 amino acids [38]. In addition, both Pax3 and Pax7 recognise identical consensus DNA sites in vitro [39], [40]. It is therefore likely that in adult muscle progenitor cells, there is significant overlap between Pax3 and Pax7 target gene selection, explaining the similar outcomes of infection with Pax3 and Pax7 RV and of inhibiting target gene transcription using the dominant-negative versions. Interestingly, as has been indicated by an earlier study [20], not all of the observed effects of Pax genes on myogenic regulatory factors can be attributed to changes in gene expression. Expression of either Pax3DN or Pax7DN led to decreased levels of *Myf5*, *MyoD* and *myogenin* mRNAs, consistent with the observed effects on MyoD and myogenin proteins, and indicating a requirement for Pax3/7 in postnatal expression of these factors. However, whereas expression of Pax3 or Pax7 led to increased levels MyoD protein (as measured either by immunostaining or by Western blot) there were no significant changes in levels of *MyoD* mRNA, suggesting mechanisms other than transcriptional control, such as increased protein stability, are also operating.

We also analysed Pax3 and Pax7 function in non-myogenic NIH 3T3 fibroblasts, which do not express these *Pax* genes, to determine whether their effects on proliferation rate and cell size were dependent on the myogenic program. In contrast to the ability of ectopic PAX3-FOXO1A [34], Pax3 and Pax7 were unable to induce each other, or myogenic proteins, in NIH 3T3 fibroblasts maintained under expansion conditions, as reported elsewhere [41] (although specific culture conditions can produce limited activation of the myogenic program after Pax3 infection [41]). Pax3 has also been reported to be unable to initiate myogenesis in the human Saos-2 osteosarcoma cell line [42] and mouse endothelial cells line BEND3 [41], and Pax7 is unable to activate myogenesis in the C3H10T1/2 multipotent mesenchymal cell line [43]. In mesenchymal Saos-2 cells however, constitutive Pax3 expression was reported to result in a significant reduction in cell size accompanied by changes in cell morphology [42]. In our experiments using NIH 3T3 fibroblasts, constitutive expression of Pax3 or Pax7 resulted in decreased cell size and increased proliferative rate, and expression of Pax3DN or Pax7DN resulted in increased cell size, decreased proliferative rate, and not uncommonly, doubling of nuclei. We do not exclude the possibility that off-target transcriptional effects contribute to the Pax3DN/Pax7DN cell phenotype. However, the effects of constitutively expressing Pax3 or Pax7 demonstrate that these factors are capable of modulating cell morphology and proliferative rate independently of their ability to regulate expression of muscle-specific genes. The recent observations that Pax3 can control components of the FGF signaling pathway provides a possible mechanism for how ectopic Pax3 may influence cell division [44].

In melanocyte precursors, Pax3 simultaneously activates expression of Mitf, a transcription factor critical for melanocyte development, and competitively inhibits Mitf binding to the dopachrome tautomerase enhancer which is required for melanin synthesis by fully-differentiated melanocytes, therefore causing cells to commit to differentiation but yet remain undifferentiated until the repressive Pax3 signal is relieved [45]. We postulate that Pax3 and Pax7 play a similar role in myogenic cells, such that activation of a transcriptional target or targets is required for progression to differentiation but differentiation is moderately repressed by promoting proliferation. Consistent with this idea, *Pax7^−/−^* satellite cells are specified but exhibit marked defects in their ability to both proliferate and differentiate [15], [17], [18]. In comparison with Pax7, Pax3 appears to be less critical for postnatal myogenesis, but recent studies have provided evidence for a role in regulating a population of interstitial myoblasts that persists in Pax7-null muscle [18]. Our results suggest that any transient expression of Pax3 with Pax7 in proliferating satellite cell-derived myoblasts [11]–[13] may function to amplify transcription of common target genes.

Our findings suggest that Pax3 and Pax7 simultaneously drive proliferation of satellite cell-derived myoblasts and keep them poised for differentiation in response to appropriate environmental cues. The ability to maintain lineage-commitment of tissue-specific stem cells whilst allowing their population expansion is a necessary aspect of preserving the integrity of differentiated adult tissues.
